# A Tablet-Based Tool for Care During Labor+Attention to System Requirements

**DOI:** 10.9745/GHSP-D-19-00384

**Published:** 2019-12-23

**Authors:** Stephen Hodgins

**Affiliations:** a Editor-in-Chief, Global Health: Science and Practice Journal, and Associate Professor, School of Public Health, University of Alberta, Edmonton, Alberta, Canada.

## Abstract

Evidence on using a tablet-based labor decision-support tool suggests the potential for improved practices in labor management. Further rigorous study on these tools is needed to assess the improvements in labor care and outcomes as well as the system requirements needed to achieve such improvements.

See related article by Sanghvi et al.

Over the past decade, it has been heartening to see marked increases in institutional births in many countries. However, this increase has not consistently been associated with corresponding declines in maternal and perinatal deaths.^1^^–^^3^ In many countries, intrapartum stillbirths and very early newborn deaths remain distressingly common. Safe, supportive, vigilant, and well-coordinated care during labor could significantly reduce the burden of such deaths.

## USING THE PAPER PARTOGRAM TO AID LABOR DECISION MAKING

The partogram is a single-page, graphic record used to document key clinical information on the mother and fetus during labor and is intended to be an aid to clinical decision making. Although the World Health Organization no longer recommends using the 4-hour action line (based on 1 cm/hour increase in dilatation through the active phase of labor), it does endorse using the partogram for monitoring the well-being of the mother and newborn and for identifying risks of adverse outcomes.^4^ In principle, as designed, partogram use might be expected to contribute to improved outcomes, mediated through the following:

- Improvement of practices that can affect placental perfusion
- More timely identification of complications or risk states and initiation of appropriate action, including transfer or referral
- Better documentation and sharing of information between involved health workers on care given and status of the mother and newborn, at handover or referral

However, the available evidence for such an effect has been disappointing. A Cochrane review by Lavender et al. (2018)^5^ found no clear evidence for an association between partogram use and improved practices or outcomes, although they offered more nuanced conclusions in a realist review on the same question.^6^ From this review, they concluded that to achieve improved outcomes:

- The partogram and all associated equipment (e.g., manometers for taking blood pressure) need to be reliably available
- Staffing needs to be adequate for patient load
- Clinical management needs to provide ongoing, committed support for partogram use and associated labor care practices, including regular audit and feedback

In short, even clinically sound, well-designed job aids cannot be expected on their own to improve practices and outcomes. Indeed, deficiencies in the use of the original paper-based partogram are well known. In many instances, they are filled in after the fact, information recorded may be inaccurate, and they are often not used for decision making. Even if they are used for decision making, there has tended to be excessive emphasis on rate of cervical dilatation.^6^^,^^7^

Even clinically sound, well-designed job aids cannot be expected on their own to improve practices and outcomes.

## USING THE TABLET-BASED PARTOGRAPH

The article by Sanghvi et al.^8^ in this issue of GHSP reports on a study conducted in Kenya that attempted to address some of these known constraints on the effectiveness of partogram use. This study examined the use of a tablet-based documentation/decision-support tool with the following key features:

- Clinical information can only be entered in real-time, not after the fact.
- Information entered into the tablet can be monitored by a supervisor, either on- or off-site.
- Automated prompts are given, encouraging supportive practices (e.g., continued ambulation, presence of a labor companion, taking fluids and food).
- Audible alarms/triggers remind the health worker on timing for reassessment and indicate if, based on the algorithms in the app, criteria are met suggesting a risk or complication requiring action. (As the authors note, when guidelines for care in labor are further revised, the algorithms used in this tool can be easily reprogrammed.)

Participants in both intervention and comparison arms were given a 2-day training on care during labor, including partogram use. Those in the intervention arm were given an additional 1-day training on the tablet-based tool, which, in the intervention arm, was subsequently used in place of the paper partogram.

The intervention was well received by participating health workers, and the study provides suggestive evidence for improved practices. However, as the authors acknowledge, there are issues with the design and implementation of the study that preclude definitive claims for impact on health outcomes. First, the study sites in the intervention and comparison arms were not well matched: the intervention arm had 2 tertiary-level hospitals with large patient volumes plus 4 health centers offering basic emergency obstetric and newborn care (BEmONC), and the comparison arm had 4 relatively lower-volume comprehensive emergency obstetric and newborn care hospitals and 2 health centers offering BEmONC. Second, compared with sites in the intervention arm, a significantly lower proportion of health workers in the comparison arm received the 2-day training. Furthermore, data on performance in the comparison arm were more limited because data were drawn only from the completed partograms.

The intervention was well received, and the study provides suggestive evidence for improved practices.

The evidence from this and similar studies is not yet sufficient to recommend large-scale adoption.^9^^–^^11^ Nevertheless, a strong case can be made for further rigorous study to better characterize what improvements can be achieved in care during labor using such tools and what system requirements must be met to achieve such improvements (such as those identified by Bedwell et al.^6^).
